# Accelerated Solvent Extraction of Antioxidant Compounds from Gardeniae Fructus and Its Acetylcholinesterase Inhibitory and PC12 Cell Protective Activities

**DOI:** 10.3390/foods10112805

**Published:** 2021-11-15

**Authors:** Yiling Fan, Xueying Li, Lan Ding, Weiying Zhou, Guangzhi Xu, Yan Wang, Youzuo Zhang, Qinxue Ni

**Affiliations:** 1 Food and Health College, Zhejiang Agriculture and Forestry University, Hangzhou 311300, China; 201824070104@stu.zafu.edu.cn (Y.F.); 201920030215@stu.zafu.edu.cn (X.L.); 202024070108@stu.zafu.edu.cn (W.Z.); guangzhi@zafu.edu.cn (G.X.); aliceyan@zafu.edu.cn (Y.W.); yyzzhang2002@zafu.edu.cn (Y.Z.); 2 Agricultural and Forestry Technology Extension Center of Lin’an, Hangzhou 311300, China; ladinglan@zjnm.cn; 3 Zhejiang Provincial Key Laboratory of Resources Protection and Innovation of Traditional Chinese Medicine, Zhejiang Agriculture and Forestry University, Hangzhou 311300, China

**Keywords:** Gardeniae fructus, accelerated solvent extraction, characteristic compounds, antioxidant, acetylcholinesterase inhibitory, PC12 cell

## Abstract

Gardeniae fructus is a common neuroprotective medicinal food in China, however the extraction efficiency and mixture activities are rarely mentioned. In this study, accelerated solvent extraction (ASE) parameters were optimized by a response surface methodology to extract antioxidants from Gardeniae fructus. Neuroprotective activity was evaluated using H_2_O_2_ and amyloid-β_25–35_ peptide-treated PC12 cells. By comparing with three other extract methods (i.e., heated refluxing extraction (HRE), ultrasound-assisted extraction (UAE), microwave-assisted extraction (MAE)), it was found that the yield (35.10%), total iridoids (27.69%), total flavonoid (6.12%) content, antioxidant activities (IC_50_ on DPPH, 164.46 µg/mL; FRAP value 4703.54 μmol/L), and acetylcholinesterase inhibitory ability (IC_50_ 92.58 µg/mL) of ASE extract under the optimal condition (150 °C temperature, 10 min static time, 60% ethanol, 2 extract cycles) were significantly higher than other extract methods. The strongest ability to protect PC12 cells from damage was also present in ASE extract, as evidenced by decreasing lactate dehydrogenase and malondialdehyde levels, elevating superoxide dismutase and glutathioneperoxidase activities. Compositional analysis indicated that the extremely high crocetin level in ASE extract (1.30 μg/mg) may offer great potential. Our results indicated that ASE is a proper extraction method that could offer great potential for finding the neuroprotective ability of Gardeniae fructus for the treatment of AD.

## 1. Introduction

*Gardenia jasminoides* Ellis is an evergreen tree with fragrant white flowers and red-brown fruits in the Rubiaceae family, distributed in multiple areas in Asia. Gardeniae fructus, the desiccative ripe fruits of this plant, were applied in china for centuries to extract natural colorants and as an important traditional herbal medicine. In Chinese Pharmacopoeia (2010), it is cold in nature and bitter in taste, with the effects of discharging fire, eliminating vexation, protecting the liver, promoting choleretics, causing diuresis, cooling blood, etc. In recent decades, research has also confirmed several pharmacological actions of Gardeniae fructus, such as antioxidant [1], anti-inflammatory [2], antidiabetic [3], anti-cancer [4], treating liver disease [5], etc.

Among the research on the various pharmacological activities of Gardeniae fructus, nowadays, a lot of attention is especially paid to their neuroprotective effects, such as anti-dementia [6], anti-depression [7], anxiolytic [8], hypnotic [9], improving cognition [10], memory improvement [11], and other brain function disorders [12]. All of these findings sufficiently showed that Gardeniae fructus has a good potential for protecting the nervous system. The possible therapeutic mechanisms mainly include preventing apoptosis of neurons and the ability of antioxidation [13]. Oxidative damage caused by excessive ROS production has received substantial attention with regards to neurodegenerative diseases and has been proposed as a contributing factor to the pathophysiology [14,15]. Thus, antioxidants have been numerously tested for their efficacy to slow down the progressive deterioration in neurodegenerative diseases [16]. 

Phytochemical studies on Gardeniae fructus have resulted in several kinds of secondary metabolites, including iridoid glycosides (e.g., geniposide, genipin, geniposidic acid, shanzhiside), carotenoids (e.g., crocetin, crocin I, crocin II), flavonoids (e.g., rutin, quercetin), saponins, polysaccharides, and phenolic acids (e.g., chlorogenic acid, ursolic acid) [17,18]. The main metabolites reported as responsible for the neuroprotective effect are geniposide and crocin [19]. However, Zhang et al. [20] compared the functions of learning and memory improvement and neuroprotection of the crude *Gardenia jasminoides* Ellis extracts (GJE) and geniposide on chronic cerebral ischemia model rats, and concluded that GJE has a better effect on improving chronic cerebral ischemia than geniposide. The diverse chemical components in Gardeniae fructus, that lead to the multi-targeted advantage or even the synergy between compounds, may explain this phenomenon, and hopefully may lead to breakthroughs for the complicated and closely related diseases, such as functional brain disorders. Therefore, searching for extracts from Gardeniae fructus with higher potency and more neuroprotective compounds will be worthwhile.

The extraction of active compounds from Gardeniae fructus is usually performed using conventional methods, such as maceration extraction, refluxing extraction, and ultrasonic extraction [21,22], which have the drawbacks of being solvent- and time-consuming and tedious to perform. Accelerated solvent extraction (ASE), as an advanced extraction technique, has several advantages, including fast sample analysis, easy automatic operation, low required solvent volume, low risk of exposure to solvents, and easy maintenance of samples in an oxygen- and light-free environment. Furthermore, ASE also allows the operator to control the temperature, pressure, extraction time, and number of extractions, which can increase the extraction efficiency from plants. Thus, ASE not only improves the extraction yield, but also decreases solvent and time consumption, and is one of the most promising alternative methods for the isolation of components from plant raw materials.

In this paper, we focus on the optimization and comparison between ASE techniques and other conventional extraction methods (i.e., heated refluxing extraction (HRE), ultrasound-assisted extraction (UAE), and microwave-assisted extraction (MAE)) to maximize the antioxidant capacity of the extracts from Gardeniae fructus. Then, total iridoid (TI), total crocin (TC), and total flavonoids (TF), along with six characteristic compounds (i.e., geniposidic acid, geniposide, genipin, crocin-2, crocin-1, and crocetin), were quantitatively determined by spectrophotometer analyses and HPLC methods. Moreover, in order to evaluate the neuroprotective activity and the possible mechanism of Gardeniae fructus extracts, the anti-acetylcholinesterase activity and protection effect on pheochromocytoma cells (PC12 cells) from H_2_O_2_- and Aβ_25–35_ peptide-induced neuronal cell damage were investigated.

## 2. Materials and Methods

### 2.1. Plant Material and Sample Preparation

The Gardeniae fructus meal was supplied by Zhejiang Jiaozhi Technology Co., Ltd. (Hangzhou, China). Briefly, the fruits of *Gardenia* *jasminoides* Ellis were harvested at full maturity in Zhejiang province of China. The fruits were washed, drained, and enzyme-inactivated by streaming (120 °C for 20–30 min) and then dried in the sun. The dried fruits were peeled off and defatted by an Oil Expeller (YJY-Z350, Pinyang, Hubei, China). The samples were then milled into a powder of 40-mesh particle size.

### 2.2. Chemicals and Reagents

Rutin (purity ≥ 98%), geniposidic acid (≥99%), geniposide (≥98%), genipin (≥98%), crocin-1 (≥98%), and huperzine A (≥98%) were purchased from Shanghai Ronghe Pharmaceutical Technology Co., Ltd., Shanghai, China. Crocin-2 (≥98%) and crocetin (≥98%) were provided by Shanghai Macklin Biochemical Co., Ltd., Shanghai, China. Acetonitrile, water, and methanol of HPLC-grade, and acetylcholinesterase (AChE), 3-(4,5-dimethyl thiazol-2-yl)-2,5-diphenyl tetrazolium bromide (MTT), hydrogen peroxide (H_2_O_2_), and amyloid-β_25–35_ peptide, were purchased from Sigma-Aldrich (St. Louis, MO, USA). Dulbecco’s modified Eagle’s medium (DMEM), fetal bovine serum (FBS), trypsin, 0.25% trypsin-EDTA, and penicillin/streptomycin mixture were purchased from Gibco-BRL (Grand Island, NY, USA). The measurement kits formalondialdelyde (MDA, Cat. No. A003-1), lactate dehydrogenase (LDH, Cat. No. A020-1), superoxide dismutase (SOD) (Cat. No. A001-1), glutathione peroxidase (GSH-Px, Cat. No. S0058), and the reagents used for enzyme assays were obtained from Nanjing Jiancheng Bioengineering Institute (Nanjing, Jiangsu, China). All other chemicals and solvents were of analytical grade from commercial suppliers in China.

### 2.3. Accelerated Solvent Extraction (ASE) Procedure and Experimental Design by RSM

The accelerated solvent extraction of antioxidants from Gardeniae fructus was performed with a DIONEX extractor (Dionex ASE^®^300, Dionex Crop., Sunnyvale, CA, USA). Gardeniae fructus meal powder (10 g) was previously mixed with diatomaceous earth and then placed in a 34 mL Dionex (ASE 300) stainless-steel cell and was extracted under 1500 psi by using conditions obtained from the response surface methodology (RSM)-guided experimental design.

A Box-Behnken design (BBD) method was employed to study the optimum condition of four factors, namely extraction temperature (°C, *X*_1_), static time (*X*_2_), ethanol concentration (% *w*/*w*, *X*_3_), and number of extraction cycles (*X*_4_). Based on the previous single-factor experiments, each variable was set at three levels. The BBD had five of the factorial points at the center of the design, for a total of twenty-nine experiments, which were performed in random order to avoid systematic error.

The second-order polynomial model equation for the response was as follows:

$$Y=\beta_{0}+\sum_{i=1}^{4}\beta_{i}X_{i}+\sum_{i=1}^{4}\beta_{ii}X_{i}{}^{2}+\sum_{i=1}^{3}\sum_{j=i+1}^{4}\beta_{ij}X_{i}X_{j}$$

where *Y* represents the response variables, *β*_0_ is a constant, and *β_i_*, *β_ii_*, and *β_ij_* are the linear, quadratic, and interactive coefficients, respectively. *X_i_* and *X_j_* are the levels of the independent variables. The selected response variable (*Y*) was the antioxidant activity, represented by diphenyl-2-picrylhydrazyl (DPPH) scavenging ability.

### 2.4. Other Extract Methods

Heated refluxing extraction (HRE) was carried out in a cooled condenser and a round-bottom flask of 250 mL. A portion of 10 g of powder of Gardeniae fructus was double extracted with 60% ethanol-aqueous solution (150 mL) under reflux in a water bath at 80 °C for 3 h.

Ultrasound-assisted extraction (UAE) was carried out in an ultrasonic cleaning bath (KQ3200B, Kunshan Ultrasonic Instrument Co., Ltd., Kunshan City, Jiangsu, China). Gardeniae fructus meal powder (10.0 g) was placed into a conical flask (250 mL) with 150 mL of 60% ethanol-aqueous solute before being placed in an ultrasonic bath (100 W, 60 °C) and double extracted for 1 h.

Microwave-assisted extraction (MAE) was carried on a WP700TL 23-K5 microwave-assisted extraction unit (Glanz Group Co., Ltd., Foshan, Guangdong, China). Gardeniae fructus meal powder (10.0 g) was mixed with 150 mL of 60% ethanol-aqueous solute in a 250 mL conical flask, and then the mixture was soaked for 1 h before being irradiated under microwave heating (500 W) for 15 min. The same sample was extracted twice.

After extraction, each resulting extract was combined, filtered, evaporated, and then lyophilized. The extraction yield of each extract method was calculated as:

Yield % = (mass of extract/mass of dry raw material) × 100(1)

### 2.5. Determination of Total Iridoids (TI), Total Crocin (TC), and Total Flavonoids (TF) Content and Antioxidant Activity

The content of total iridoids (TI) was determined by the ultraviolet spectrophotometer method with geniposide as a standard [23]. Briefly, the absorbance of the standard solution at 238 nm was measured against a blank of 50% methanol. A standard curve with absorbance as the horizontal axis and geniposide concentration as the vertical axis was charted (y = 0.0103x + 0.0352, *r* = 0.9992). The amount of TI in Gardeniae fructus extracts was calculated as a geniposide equivalent from the standard curve.

The total crocin (TC) content was measured with crocin-1 as a standard [24]. The absorbance of crocin-1 stock solution at 440 nm was measured. A standard curve was established using absorbance as the horizontal axis and crocin-1 concentration as the vertical axis (y = 0.2675x + 0.0194, *r* = 0.9997). The TC content was calculated as a crocin-1 equivalent from the standard curve.

The total flavonoid (TF) content was determined using the aluminum chloride colorimetric method described previously [25], with rutin as a standard. A standard curve with absorbance as the horizontal axis and rutin concentration as the vertical axis was charted (y = 10.2243x + 0.0051, *r* = 0.9998). The amount of TF was calculated as a rutin equivalent from the standard curve.

The DPPH free radical scavenging activity was measured using the method described previously [26], with some modifications. Samples (2.0 mL) of different concentrations were mixed with 2.0 mL DPPH solution and reacted for 1 h. The absorbance at 517 nm was measured, with methanol as a blank. The DPPH radical scavenging rate (%) was calculated in the following way: % radical scavenging rate = (A_blank_ − A_sample_)/A_blank_ × 100. The results were expressed as IC_50_ of DPPH radical scavenging.

The FRAP assay was performed according to the procedures of Benzie et al. [27] with minor modifications, as described previously [28]. Gardeniae fructus extracts were prepared at the same concentration (250.0 μg/mL). The FRAP parameter was defined as the concentration of antioxidants having a ferric reducing ability equivalent to that of 1 µmol of FeSO_4_.

### 2.6. Acetylcholinesterase (AChE) Inhibition Assay

AChE inhibitory activity was determined by the Ellman assay [29] with minor modifications. The Ellman assay was performed in 96-well plates. Briefly, 100 μL of phosphate buffer (100 mM sodium phosphate buffer, pH 8.0), 100 μL samples (at different concentration), and 10 μL of AChE (0.1 U/mL) were added to the wells. After a pre-incubation at room temperature for 20 min, 40 μL of substrate solution (equal volume of 0.6 mM 5′,5-dithio-bis-(2-nitrobenzoate) (DTNB) and 0.6 mM acetylthiocholine iodide (ATCI)) was added. The plates were incubated at 37 °C for 20 min before the absorbance was measured in a synergy H1 microplate reader (BioTek, Winooski, VT, USA) at 405 nm. The AChE inhibition rate (%) was calculated in the following way: % inhibition rate = (1 − (A_3_ − A_4_)/(A_1_ − A_2_)) × 100, using the absorbance of A_1_ (sample replaced by PBS), A_2_ (sample and enzyme replaced by PBS), A_3_ (whole reaction solution), and A_4_ (enzyme replaced by PBS). Inhibitory effects were expressed as IC_50_ values, which means the concentration of samples necessary to inhibit AChE by 50%.

### 2.7. Six Characteristic Compounds of Gardeniae Fructus Analysis by HPLC

The amounts of six characteristic compounds of Gardeniae fructus, i.e., geniposidic acid, geniposide, genipin, crocin-1, crocin-2, and crocetin, were analyzed using HPLC, as described by Bergonzi et al. [30] with some modifications. The HPLC system was LC-20AT (Shimadzu, Japan), which consists of a SPD-M20A diode array detector and UV-1800 UV-Vis spectrophotometer, equipped with a reversed-phase C_18_ analytical column of 4.6 × 250 mm and 5 μm particle size (Inertsil^®^ ODS-SP). Column temperature was maintained at 35 °C. The injection volume was 10 μL. Mobile phase was 0.3% formic acid aqueous solution (A), and (B) methanol:acetonitrile (9:1). The optimized chromatographic condition was: 0~15 min, A 90~50%, B 10~50%; 15~18 min, A 50%, B 50%, 18~35 min, A 50~0%, B 50~100%; 35~40 min, A 0%, B 100%; 40~45 min, A 0~90%, B 100~10%, and a 5 min post-run was used after each analysis. The flow rate was 0.8 mL/min. The standard solution containing geniposidic acid 70.66 μg/mL, geniposide 71.66 μg/mL, genipin 183.40 μg/mL, crocin-1 70.34 μg/mL, crocin-2 70.00 μg/mL, and crocetin 39.66 μg/mL was prepared. The standard solution was filtered through a 0.45 μm filter before being analyzed by HPLC. Geniposidic acid, geniposide, and genipin were detected at 254 nm, while crocin-1, crocin-2, and crocetin were detected at 440 nm. The HPLC method evaluation was performed by the precision, repeatability, stability, and recovery test. Curves of six standard compounds using concentration as abscissa and peak area as the vertical axis were charted (Table 1).

### 2.8. PC12 Cell Culture and Treatment

The cell cultures were prepared according to the method described by Guimarães et al. [31]. Briefly, the PC12 cells, a clonal cell line derived from a rat adrenal medulla tumor (Institute of Biochemistry and Cell Biology, SIBS, CAS, Shanghai, China), were cultured in Dulbecco’s Modified Eagle’s Medium (DMEM) supplemented with 10% fetal bovine serum (FBS), 100 U/mL penicillin, and 100 μg/mL streptomycin in a humidified atmosphere composed of 95% air and 5% CO_2_. Culture medium was changed every other day. Before all experiments, aliquots of cells in the exponential growth phase were plated in 96-well poly-D-lysine-coated plates at a density of 1 × 10^5^ cells/mL (100 μL/well) and allowed to adhere for 24 h, at 37 °C, in complete DMEM medium.

In our preliminary experiment, 25~300 μmol/L of H_2_O_2_ and 5~40 μmol/L of Aβ_25–35_ peptide were treated with PC12 cells, and 100 μmol/L of H_2_O_2_ and 20 μmol/L of Aβ_25–35_ peptide were found to be the most suitable concentrations for use in the injury model, achieving 54.08% and 49.58% cell survival rates, respectively. Thus, in this study, the damage model group cells were treated with 100 μmol/L of H_2_O_2_ or 20 μmol/L of Aβ_25–35_ peptide. The sample group cells were pretreated with six concentrations of samples (6.25~100 μg/mL) for 2 h and then incubated in 100 μmol/L of H_2_O_2_ or 20 μmol/L of Aβ_25–35_ peptide for an additional 24 h at 37 °C and 5% CO_2_. The control group cells were treated with the same medium without sample, H_2_O_2_, or Aβ_25–35_ peptide.

### 2.9. Cell Viability Assay

Cell viability was estimated by the MTT assay [32]. Briefly, the cells were incubated for 24 h at 37 °C and 5% CO_2_ in 96-well culture plates at 1.0 × 10^5^ cells/well. After treatment with samples or H_2_O_2_ and Aβ_25–35_ peptide, the medium was removed and 50 μL of fresh DMEM and 10 μL of MTT (5 mg/mL) were added to the wells. The plates were incubated for 4~8 h, at 37 °C and 5% CO_2_. The supernatant was discarded and 100 μL of dimethyl sulfoxide (DMSO) was added to each well to dissolve the formazan. Absorbance was recorded at 570 nm in the microplate reader (BioTek, Winooski, VT, USA). Cell viability was normalized as relative percentages in comparison with untreated controls (100% viability).

### 2.10. Analyses for Lactate Dehydrogenase (LDH), T-SOD, GSH-Px Activity, and Malondialdehyde (MDA) Content

The PC12 cells (1 × 10^5^ cells/well in 96-well plates) were treated in the same way as described above. All samples were prepared at the concentrations of 6.25, 25, and 100 μg/mL. The supernatant was then collected and used in the assay. The LDH, T-SOD, GSH-Px activities, and MDA content were performed using the commercial assay kits according to the manufacturer’s instructions (Jiancheng Institute of Biotechnology, Nanjing, China). The LDH, SOD, and GSH-Px activity were expressed as U/L, U/mL, and U respectively, while MDA content was expressed as nmol/mL.

### 2.11. Statistical Analysis

All experimental data were analyzed using analysis of variance (ANOVA). Analysis of significant differences was performed using Duncan’s new multiple range method. Data are expressed as average value ± standard deviation. All experiments were carried out in triplicate. “SPSS 19.0” statistical software (International Business Machines Corporation, New York, NY, USA) was used for data analysis.

## 3. Results and Discussion

### 3.1. Optimization of ASE Parameters

The Box-Behnken design (4^3^ factorial), with a total of twenty-nine experiments, and the actual and predicted data obtained according to the RSM design are listed in Table 2. The ANOVA result for the model of ASE conditions is shown in Table 3.

As the results show, the model F-value was 20.42 and the model *p*-value was less than 0.0001, indicating the goodness of fit of the model. The correlation coefficient (*R*^2^ value) was 0.9533, indicating that the calculated model could explain 95.33% of the variability in the response. The lack of fit value was not significant (*p* = 0.0822), confirming the validity of the model. The value of the coefficient of variation (C.V. %) was 3.14%, suggesting that the model is reproducible. The adjusted coefficient of determination (Adj. R^2^) was 0.9066 for the polynomial model. Therefore, the model can be used as an estimate of a trend, to be able to predict the influence of the extraction parameters on the antioxidant ability of the extracts.

In addition, the significance of the regression coefficients of intercept, linear, quadratic, and interaction terms of the model were determined by the F-value and the *p*-value. Values of *p* < 0.01 indicated that the model term was highly significant. As shown in Table 2, three linear (*X*_1_, *X*_3_, and *X*_4_), two quadratic (*X*_1_^2^ and *X*_3_^2^), and one interaction parameters (*X*_1 × 4_) were highly significant model terms. Moreover, the influence order of the single factor was temperature (*X*_1_) > ethanol concentration (*X*_3_) > extraction cycles (*X*_4_) >static time (*X*_2_).temperature (X_1_) > ethanol concentration (X_3_) > extraction cycles (X_4_) > static time (X_2_).

The coefficients of independent variables determined for the second-order polynomial model are shown in the following equation (in terms of coded levels):

(2) $$Y=88.27+9.32X_{1}+1.20X_{2}-3.69X_{3}+2.57X_{4}–\;1.11X_{1}{X\;}_{2}+2.18X_{1}{X\;}_{3}-4.10X_{1}X_{4}+0.77X_{2}X_{3}-0.26X_{2}X_{4}+1.87X_{3}X_{4}-8.34X_{1}^{2}-0.35{X\;}_{2}^{2}–\;3.88X_{3}^{2}-1.48X_{4}^{2}$$

In Equation (2), X_1_ is the temperature, X_2_ is the static time, X_3_ is the ethanol concentration, and X_4_ is the extraction cycles.

**Figure 1:**
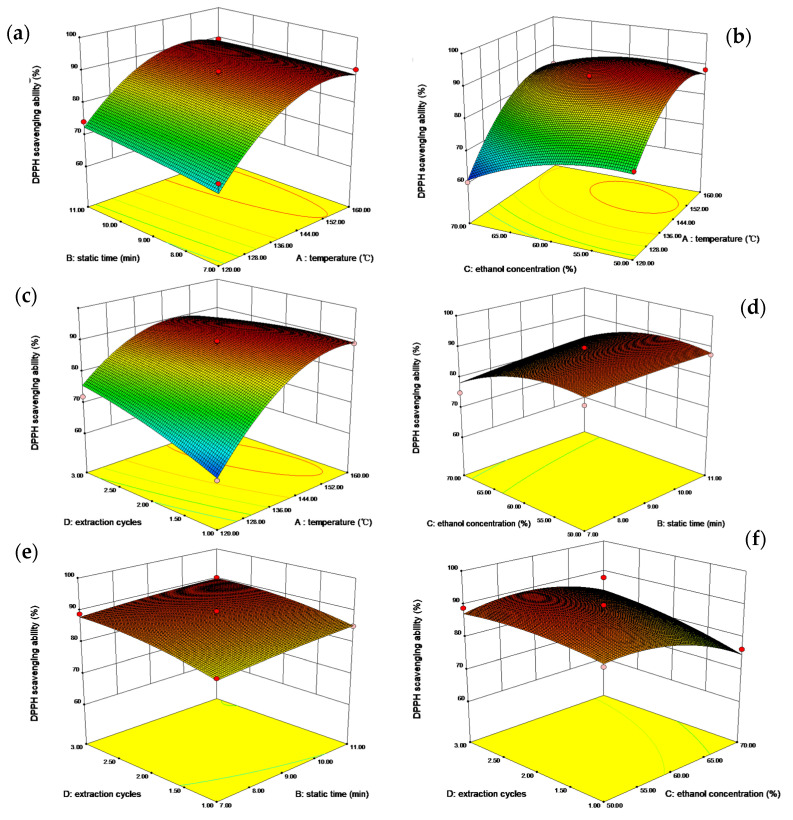
Response surface plots showing effects of the ASE parameters on the antioxidants’ extraction from Gardeniae fructus. The model was constructed by varying two variables within the experimental range under investigation and holding the other two variables at the “0” level: (**a**) varing temperature and static time; (**b**) varing temperature and ethanol concentration; (**c**) varing temperature and extraction cycles; (**d**) varing static time and ethanol concentration; (**e**) varing static time and extraction cycles; (**f**) varing ethanol concentration and extraction cycles.

The three-dimensional response surfaces of the independent variables are shown in Figure 1. Figure 1a–c showed the effects of temperature with each of the three other factors on the antioxidant activity of extracts. The results implied that the temperature was of greater significance than the other three variables. In all cases, the antioxidant ability of extracts significantly increased with the temperature, until reaching a maximum value at about 150 °C, and then as the temperature continued to increase, the response showed a slightly downward trend. This result is consistent with the study by Andrew et al. [33]. It may be explained by the fact that under the conditions below 150 °C, higher temperature may promote the dissolution of antioxidants into the extraction solvents, however, for temperatures exceeding 150 °C, some antioxidants are heat-sensitive and degraded by high temperatures. Figure 1a,d,e show that the influence of static time was not as significant as that of the other three factors. The antioxidant ability did not change significantly when prolonging the static time. Ethanol concentration also had a great influence on the extraction efficiency, as shown in Figure 1b,d,f. When the ethanol concentration reached about 60%, the response reached the highest value. This may be attributed to the fact that 60% ethanol was the closest polarity to the antioxidant substances in Gardeniae fructus. In Figure 1c,e,f, a trend towards a higher response was obtained by increasing the number of extraction cycles. However, the trend became flat as the extraction cycles kept increasing. The possible reason for this is the accumulation of the antioxidants in the extracts.

The fitted model was applied to predict the optimum values of the four variables. The optimized results showed that the most suitable conditions for extracting the ASE antioxidants from Gardeniae fructus were as follows: temperature (149.81 °C), static time (10.32 min), ethanol concentration (57.15%), and number of extraction cycles (1.95). The suitability of the model equation for predicting the optimum response values was tested using the selected optimal conditions (adjusted according to the actual situation: temperature 150 °C, static time 10 min, ethanol concentration 60%, number of extraction cycles 2), and the practical value of the DPPH scavenging ability of the extract was 90.71% ± 0.82%, which was consistent with the predicted value (91.41%).

### 3.2. Comparison of Extract Methods on the Yield, Active Components’ Content, and Characteristic Compounds from Gardeniae Fructus

#### 3.2.1. Gardeniae Fructus Extracts’ Yield and Active Components’ Contents

Extraction is an important step for the recovery and isolation of bioactive phytochemicals from plant materials. In the present study, the yield and active components’ content of Gardeniae fructus extracts, which related to the different extract methods, i.e., HRE, UAE, MAE, and ASE, were observed (Table 4). Results showed that extract methods had a significant effect on the recovery of the active substances, and the yield decreased in the following order: ASE (35.10%) > HRE (28.97%) > UAE (27.91%) > MAE (16.73%). ASE had the highest extraction efficiency with the shortest time, which could be attributed to the use of pressure vessels, which greatly facilitate the penetration of the extracting solvent in a liquid form even when working above its boiling temperature, through the food matrix. The extraction yield between HRE and UAE had no significant difference. However, MAE showed a much lower extraction yield than the other extraction methods. We suppose that microwave irradiation may cause internal over-heating, which in an aerobic environment may lead to carbonization and then other reactions, such as isomerization and/or degradation of compounds in Gardeniae fructus [34].

Quantification of total active components showed an interesting result, that pressured solvent extraction also obtained a relatively higher content of iridoids and flavonoids. As shown in Table 4, ASE extract possessed the highest concentration of iridoids and flavonoids, followed by HRE, and finally UAE and MAE. This is in line with the conclusion of [35], that elevated temperature and high pressure facilitate the release of flavonoids and other bioactive compounds from the plant matrix. However, the content of total crocins in the different Gardeniae fructus extracts was exactly the opposite. The content decreased in the following order: UAE > MAE > HRE > ASE, and a great loss of total crocins was observed in ASE extract, which may be due to the degradation of crocin by heating because of their unstable characteristics [36].

#### 3.2.2. HPLC Analysis of Six Characteristic Compounds in Different Gardeniae Fructus Extracts

Iridoids (i.e., geniposide, genipin, geniposidic acid) and carotenoids (i.e., crocetin, crocin-1, crocin-2) are the most effective constituents in Gardeniae fructus [37]. Thus, these six characteristic compounds’ content in different extracts were compared by the HPLC method, with iridoids detected at 254 nm and crocins detected at 440 nm. The amount of each compound in different extracts was calculated according to the standard curves (Table 5). The contents of the detected compounds were in the order of geniposide > crocin-1 > geniposidic acid > crocin-2 > genipin > crocetin.

Table 5 indicates that geniposide is the main compound in Gardeniae fructus extracts, and the content in extracts by different extract methods varied from 155.27 to 169.41 μg/mg, while genipin was hardly detected in the samples (0.41~0.71 μg/mg). However, the comparison of different extract methods found that, contrary to the result of TI contents, the content of geniposide in ASE extract was much lower than in the other extracts (*p* < 0.05). However, genipin increased a lot by ASE, which may be due to the breakdown of glycosides into the corresponding aglycones under the high extraction temperature of ASE.

The detachment of aglycon was also found in crocins of ASE extract. As a good source of natural pigments, crocins are the important compounds in Gardeniae fructus because of their board prospect of applications and pharmacological activities, with crocin-1 as the main crocin (54.10~99.08 μg/mg in Gardeniae fructus extracts). However, as is known, crocins are heat-sensitive, and can easily be removed from their aglycon under high extraction temperatures. As shown in Table 5, ASE extract possessed significantly lower contents of both crocin-1 and crocin-2 compared to the other extract methods. Consequently, crocetin increased dramatically in ASE extract (1.30 μg/mg), more than ten times higher than other extracts.

The level of geniposidic acid changed slightly between different extract methods, ranging from 1.77 to 2.14 μg/mg.

### 3.3. Effects of Extract Methods on the Antioxidant and Acetylcholinesterase (AChE) Inhibitory Abilities

**Figure 2:**
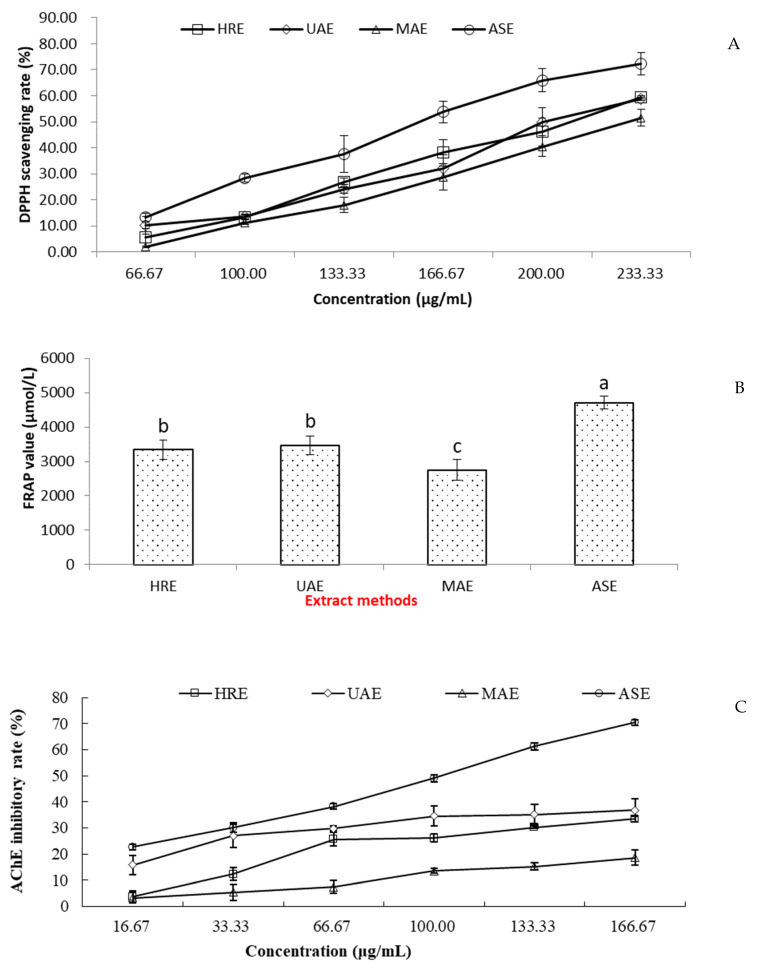
Antioxidant and AChE inhibitory activities of the different Gardeniae fructus extracts. (**A**) DPPH scavenging ability, (**B**) FRAP assay, and (**C**) AChE inhibitory ability. HRE: heated refluxing extraction; UAE: ultrasound-assisted extraction; MAE: microwave-assistedextraction; ASE: accelerated solvent extraction. Note: a–c, in (**B**) mean significant difference between the extract methods (*p* < 0.05).

The antioxidant activities of Gardeniae fructus extracts were evaluated by the DPPH radical scavenging test and FRAP assay. The reduction of the DPPH absorbance at 517 nm after 1 h of incubation was measured with different concentrations of the extracts (Figure 2A). ASE extract showed a higher DPPH scavenging rate at all tested concentrations and also a significantly lower IC_50_ (151.35 ± 4.86 µg/mL) compared to HRE (206.14 ± 7.78 µg/mL), UAE (210.28 ± 10.66 µg/mL), and MAE (234.26 ± 16.18 µg/mL), which indicated that ASE extract had the highest DPPH radical scavenging ability. The FRAP value of different extracts followed the same trend as DPPH scavenging ability. The ability of Gardeniae fructus extracts to reduce Fe^3+^ to Fe^2+^ by different extract methods decreased in the following order: ASE > UAE > HRE > MAE (Figure 2B).

The dose-dependent AChE inhibitory ability of the Gardeniae fructus extracts by different extract methods was observed in the present study, and the results are shown in Figure 2C. Among the four extract methods, the most potent inhibition appeared to be present in the ASE extract (IC_50_ 92.58 µg/mL), while the positive control huperzine A had an IC_50_ of 6.93 µg/mL. The inhibitory rates of the other three extracts also showed a dose-dependent relationship, yet, the levels were all lower than 50% at all the test concentrations, indicating that Gardeniae fructus extracts by HRE, UAE, and MAE possessed slight AChE inhibitory abilities.

Thus, as the results show, among the four extract methods, the highest extract yield, content of TI, TF, and consequently, the most potent antioxidant and AChE inhibitory abilities, appeared to be present in the ASE extract. This may be due to its high temperature and pressure which may facilitate the release of bioactive compounds from Gardeniae fructus, as well as the formation of various bioactive thermal degradation compounds.

### 3.4. Gardeniae Fructus Extracts Protect PC12 Cells against H_2_O_2_- or Aβ_25–35_-Induced Cytotoxicity

**Figure 3:**
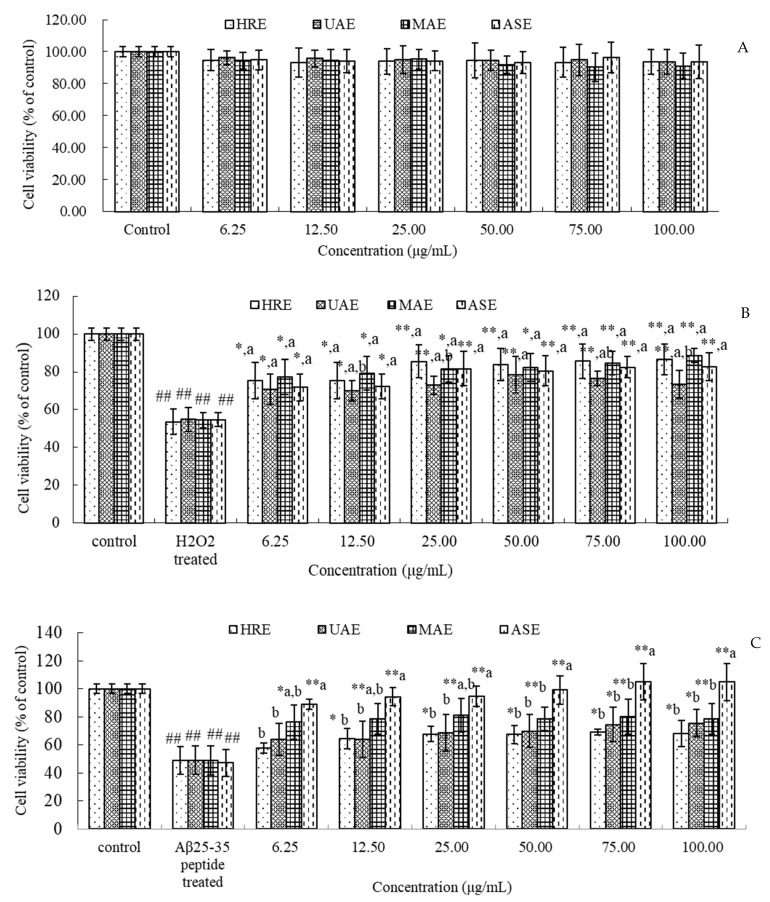
Effects of Gardeniae fructus extracts on the cell viability of PC12 cells in (**A**) basic condition, (**B**) H_2_O_2_-induced, or (**C**) Aβ_25–35_-induced toxicity. PC12 cells were pre-incubated with extracts (6.25–100 μg/mL) for 2 h prior to treatment with 100 μmol/L of H_2_O_2_ or 20 μmol/L of Aβ_25–35_ peptide for 24 h. After the treatment, cell viability was determined by MTT analysis (n = 4). Data were shown as means ± SD. ## *p* < 0.01 compared to control, * *p* < 0.05, ** *p* < 0.01 compared to H_2_O_2_- or Aβ_25–35_ peptide-treated groups. a,b mean significant difference between four different extract methods (*p* < 0.05).

PC12 cells were used to evaluate the neuroprotective ability of Gardeniae fructus extracts. The cytotoxicity of different Gardeniae fructus extracts were firstly tested by the MTT assay. Values are expressed as % of control, with the value obtained for untreated cells taken as 100%. As Figure 3A shows, there was no considerable growth inhibition in PC12 cells following 24 h of Gardeniae fructus extracts’ treatment (*p* > 0.05). The results indicate that all Gardeniae fructus extracts had no poisonous effect at concentrations of 6.25 to 100 μg/mL for 24 h on PC12 cells. Therefore, this concentration range was chosen for the further study.

To determine the protective effects of different Gardeniae fructus extracts on H_2_O_2_- or Aβ_25–35_ peptide-induced cytotoxicity, PC12 cells were pre-incubated with extracts (6.25–100 μg/mL) for 2 h prior to treatment with H_2_O_2_ or Aβ_25–35_ peptide for 24 h. As shown in Figure 3B, H_2_O_2_ (100 μmol/L) significantly decreased the cell viability to about 54%, when compared to control (DMSO alone). However, when the cells were pre-incubated for 2 h with Gardeniae fructus extracts, the H_2_O_2_-induced PC12 cell toxicity was dose-dependently decreased by all extracts. The protective effects of different extract methods showed no significant difference (*p* > 0.05).

Figure 3C shows that all Gardeniae fructus extracts at all tested concentrations could reduce Aβ_25–35_ peptide-induced (20 μmol/L) PC12 cell toxicity, with a dose-dependent relationship. Compared to other extract methods, ASE indicated the best results (*p* < 0.05). When the PC cells were pre-incubated with ASE extract at 6.25, 12.5, 25.50, 75, and 100 μg/mL, the viability of cells increased to 88.68%, 94.32%, 94.51%, 99.11%, 105.01%, and 104.79%, respectively.

**Figure 4:**
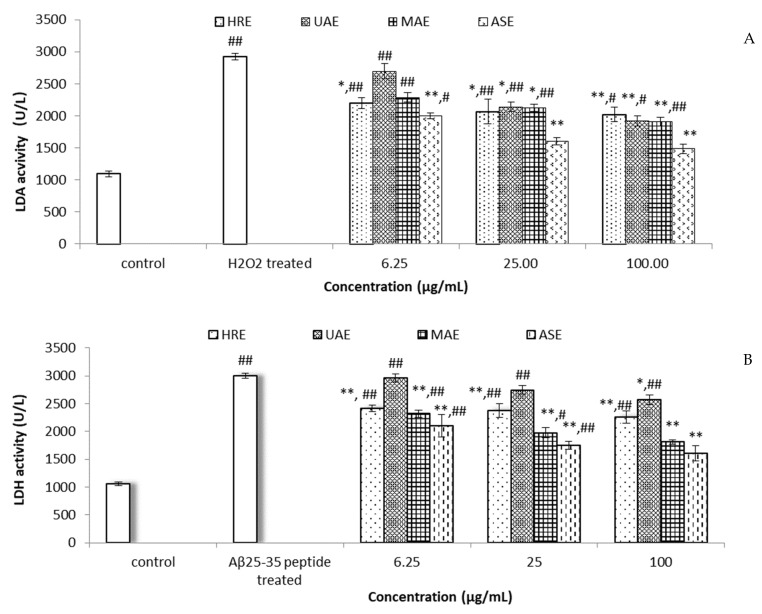
Effects of Gardeniae fructus extracts on the LDH release of (**A**) H_2_O_2_-induced or (**B**) Aβ_25–35_-treated PC12 cells. PC12 cells were pre-incubated with extracts (6.25, 25, and 100 μg/mL) for 2 h (n = 4). Data are shown as means ± SD. # *p* < 0.01, ## *p* < 0.01 compared to control, * *p* < 0.05, ** *p* < 0.01 compared to H_2_O_2_- or Aβ_25–35_ peptide-treated groups.

To further investigate the PC12 cells’ protective effect of Gardeniae fructus extracts, the LDH assay was performed. LDH, a stable cytoplasmic enzyme present inside cells, will release into the cell culture supernatant when the cell membrane is damaged [38]. Thus, the LDH activity in the supernatant could be used to access the extent of cellular damage and cytotoxicity. As shown in Figure 4, when PC12 cells were treated with H_2_O_2_ or Aβ_25–35_ peptide for 24 h, a significant increase of LDH release was observed compared to the control (*p* < 0.01). However, all Gardeniae fructus extracts could dose-dependently block the LDH leakage. Among the extract methods, ASE showed the highest protective ability on both kinds of damaged PC12 cells. At all tested concentrations (6.25~100 μg/mL), ASE could significantly reduce the cellular damage compared to the H_2_O_2_ or Aβ_25–35_ peptide treatment groups (*p* < 0.01). Furthermore, ASE pretreatment could restore the PC12 cells damaged by H_2_O_2_ and Aβ_25–35_ peptide to the control level (*p* > 0.05), from the concentrations of 25 and 100 μg/mL, respectively.

### 3.5. Gardeniae Fructus Extracts Reduced Oxidative State in H_2_O_2_ and Aβ_25–35_ Peptide PC12 Cells

It has been reported that H_2_O_2_- and Aβ_25–35_ peptide-induced PC12 cell death are partly mediated by oxidative stress [39,40]. SOD and GSH-Px are important antioxidant enzymes in mammalian cells, which play a critical role against oxidative stress-induced cell damage. To determine whether the protective effect of Gardeniae fructus extracts on H_2_O_2_- and Aβ_25–35_ peptide-induced injury were mediated by their antioxidant function, the present study examined the effects of different Gardeniae fructus extracts on the activities of SOD and GSH-Px in H_2_O_2_- and Aβ_25–35_ peptide-induced PC12 cells. Data are shown in Table 6 and Table 7. It was shown that H_2_O_2_ treatment significantly decreased the activities of SOD and GSH-Px by 65.74% and 59.12%, respectively (*p* < 0.01 vs. control). All Gardeniae fructus extracts showed the dose-dependent ability to increase the activities of antioxidant enzymes, while ASE reflected the strongest potency. The same situation occurred in Aβ_25–35_ peptide-treated PC12 cells. Aβ_25–35_ peptide treatment decreased the activities of SOD and GSH-Px by 63.66% and 69.35% respectively (*p* < 0.01 vs. control), while the damage could be restored by Gardeniae fructus extracts, especially by ASE, at the concentration of 100 μg/mL, to the control level (*p* > 0.05).

Lipid peroxidation (LPO) is one of the most important mechanisms contributing to oxidative stress. The level of malondialdehyde (MDA), an end product of lipid peroxidation (LPO), was measured to determine whether Gardeniae fructus extracts exert their protection by inhibiting ROS production. As shown in Table 8, MDA was in a low content in the control group, however 100 μg/mL of H_2_O_2_ and 20 μg/mL of Aβ_25–35_ peptide exposure for 24 h significantly increased MDA content by about 2-fold compared to the control (*p* < 0.01). However, Gardeniae fructus extracts’ pre-incubation remarkably decreased MDA content and showed a dose-dependent ability. At the concentration of 100 μg/mL, all Gardeniae fructus extracts decreased MDA content by more than 27.02% and 22.78% respectively, compared to the H_2_O_2_- or Aβ_25–35_ peptide-treated groups. Moreover, among the different extract methods, ASE showed the highest protective ability in both H_2_O_2_- and Aβ_25–35_ peptide-induced PC12 cells, which decreased MDA content by 23.04%, 30.36%, and 37.94% at 6.25, 25, and 100 μg/mL in H_2_O_2_-induced PC12 cells, and by 24.61%, 32.07%, and 42.59% in Aβ_25–35_ peptide-induced PC12 cells.

The results showed that pretreatment with Gardeniae fructus extracts significantly attenuated the oxidative damage of PC12 cells induced by H_2_O_2_ and Aβ_25–35_ peptide, as reflected by maintaining the activity of antioxidant enzymes and the content of MDA. Especially ASE, at high concentrations, could restore the damage to the control level (*p* > 0.05).

Gardeniae fructus has been commonly used for targeting neurodegenerative diseases in traditional Chinese medicine prescriptions, and also reported in many papers recently. The present study compared different extract methods, and found that ASE possessed particularly excellent neuroprotective ability. This could be due to its elevated temperature and high pressure, which facilitate the release of bioactive compounds from Gardeniae fructus by increasing diffusion rates and disrupting some of the solute–matrix interactions [41], consequently affecting the bioactivities. Degradation of bioactive compounds by microwave and ultrasound waves [42,43] may lead to the lower bioactivity of MAE and UAE extracts.

Another point worth noting was obtained by compositional analysis, showing that the content of concetin in ASE extract (1.30 μg/mg) was almost ten times higher than other extraction methods, and may offer great potential to the different bioactivities of extracts by different extraction techniques. Thus, the formation of thermal degradation compounds that have various bioactivities may also be an important reason for the increase of the bioactivity level in the ASE extract, which is worthy of further study.

## 4. Conclusions

In summary, in the present study, we optimized the ASE method to extract antioxidants from Gardeniae fructus. Based on our RSM design, optimal conditions were found to be 10 min static extraction at 150 °C, with 60% ethanol concentration under 1500 psi and two extract cycles. Surprisingly, under the optimum conditions, ASE, which possessed more potency of antioxidant activity than the other extract methods, also appeared to be the best for AChE inhibitory activity and protecting PC12 cells against H_2_O_2_- and Aβ_25–35_ peptide-induced cytotoxicity. The high content of concetin in ASE was perhaps the main reason for this. The results of this study indicated that accelerated solvent extraction is a proper extract method to obtain antioxidant compounds from *Gardenia jasminoides* Ellis fruits, and the extract offers great potential for neuroprotective ability and treatment of AD. The characteristic compounds and their possible molecular mechanisms involved require further study.

## Figures and Tables

**Table 1:** Linear equations, correlation coefficients, precision, repeatability, stability, and recovery analysis of six compounds from Gardeniae fructus (*n* = 7).

Compounds	Retention Time (min)	Detection Wavelength (nm)	Equations	*r*	Linear Range (μg/mL)	Precision, RSD%	Repeatability, RSD%	Stability, RSD%	Recovery%, RSD%
Geniposidic acid	14.522	254	Y = 10,925X + 5533.4	0.9992	1.10~70.66	1.38	1.67	2.37	98.76, 2.01
Geniposide	19.702	254	Y = 10,718X + 6062	0.9995	1.12~71.66	1.27	1.33	1.62	102.07, 1.47
Genipin	21.820	254	Y = 21,495X + 11,519	0.9997	1.84~183.40	0.97	1.05	1.17	103.21, 1.13
Crocin-1	26.475	440	Y = 33,383X + 16,746	0.9991	1.09~70.00	1.57	1.96	2.94	98.12, 2.34
Crocin-2	28.534	440	Y = 81,282X + 41,240	0.9992	1.10~70.34	1.98	2.47	3.85	98.52, 2.04
crocetin	38.042	440	Y = 33,383X + 16,746	0.9998	0.62~39.66	1.64	1.98	3.01	97.94, 2.55

**Table 2:** Box-Behnken design for optimization of the extraction of antioxidants from Gardeniae fructus by ASE and values of observed responses.

Run	Independent Variable				DPPH Scavenging Ability (%)	
	*X*_1_	*X*_2_	*X*_3_	*X*_4_	Experimental	Predicted
	Temperature (°C)	Static Time (min)	Ethanol Concentration (% *w*/*w*)	Number of Extraction Cycles		
1	−1 (120)	−1 (7)	0 (60)	0 (2)	70.73	67.95
2	1 (160)	−1 (7)	0 (60)	0 (2)	90.36	88.81
3	−1 (120)	1 (11)	0 (60)	0 (2)	74.28	72.58
4	1 (160)	1 (11)	0 (60)	0 (2)	89.47	89.00
5	0 (140)	0 (9)	−1 (50)	−1 (1)	84.99	85.91
6	0 (140)	0 (9)	1 (70)	−1 (1)	76.37	74.79
7	0 (140)	0 (9)	−1 (50)	1 (3)	88.96	87.29
8	0 (140)	0 (9)	1 (70)	1 (3)	87.83	83.66
9	−1 (120)	0 (9)	0 (60)	−1 (1)	61.68	62.46
10	1 (160)	0 (9)	0 (60)	−1 (1)	89.10	89.31
11	−1 (120)	0 (9)	0 (60)	1 (3)	72.04	75.80
12	1 (160)	0 (9)	0 (60)	1 (3)	83.04	86.23
13	0 (140)	−1 (7)	−1 (50)	0 (2)	84.91	87.29
14	0 (140)	1 (11)	−1 (50)	0 (2)	87.53	88.17
15	0 (140)	−1 (7)	1 (70)	0 (2)	75.05	78.38
16	0 (140)	1 (11)	1 (70)	0 (2)	80.74	82.33
17	−1 (120)	0 (9)	−1 (50)	0 (2)	73.40	72.60
18	1 (160)	0 (9)	−1 (50)	0 (2)	88.35	86.88
19	−1 (120)	0 (9)	1 (70)	0 (2)	60.12	60.87
20	1 (160)	0 (9)	1 (70)	0 (2)	83.78	83.86
21	0 (140)	−1 (7)	0 (60)	−1 (1)	82.91	82.41
22	0 (140)	1 (11)	0 (60)	−1 (1)	85.17	85.34
23	0 (140)	−1 (7)	0 (60)	1 (3)	88.96	88.07
24	0 (140)	1 (11)	0 (60)	1 (3)	90.18	89.95
25	0 (24)	0 (9)	0 (60)	0 (2)	89.69	88.27
26	0 (140)	0 (9)	0 (60)	0 (2)	89.86	88.27
27	0 (140)	0 (9)	0 (60)	0 (2)	87.46	88.27
28	0 (140)	0 (9)	0 (60)	0 (2)	87.41	88.27
29	0 (140)	0 (9)	0 (60)	0 (2)	86.92	88.27

**Table 3:** Estimated regression coefficients for the quadratic polynomial model and the analysis of variance (ANOVA) for the experimental results.

Factor	Estimated Coefficient	Standard Error	Degree of Freedom	Sum of Squares	Mean of Squares	*F*-Value	*p*-Value ^a^ Prob > F	
Intercept	88.27	1.16	1					
Linear								
X_1_ (°C)	9.32	0.75	1	1042.54	1042.54	155.70	<0.0001	
X_2_ (min)	1.20	0.75	1	17.40	17.40	2.60	0.1293	
X_3_ (% *w*/*w*)	−3.69	0.75	1	163.17	163.17	24.37	0.0002	
X_4_	2.57	0.75	1	79.00	79.00	11.80	0.0040	
Quadratic								
X_1_^2^	−8.34	1.02	1	450.97	450.97	67.35	<0.0001	
X_2_^2^	−0.35	1.02	1	0.78	0.78	0.12	0.7387	
X_3_^2^	−3.88	1.02	1	97.56	97.56	14.57	0.0019	
X_4_^2^	−1.48	1.02	1	14.17	14.17	2.12	0.1678	
Interaction								
X_1 × 2_	−1.11	1.29	1	4.93	4.93	0.74	0.4054	
X_1 × 3_	2.18	1.29	1	18.97	18.97	2.83	0.1145	
X_1 × 4_	−4.10	1.29	1	67.40	67.40	10.07	0.0068	
X_2 × 3_	0.77	1.29	1	2.36	2.36	0.35	0.5625	
X_2 × 4_	−0.26	1.29	1	0.27	0.27	0.040	0.8436	
X_3 × 4_	1.87	1.29	1	14.03	14.03	2.09	0.1698	
Model				1914.69	136.76	20.42	<0.0001	significant
Residual			14	93.74	6.70			
Lack of fit				85.98	8.60	4.43	0.0822	not significant
Pure error				7.76	1.94			
Cor Total				2008.43				
*R*^2^ = 0.9533, Adj. *R*^2^ = 0.9066, Pred *R*^2^ = 0.7474, Adeq precision = 15.630, C.V.% = 3.14								

^a^ *p* < 0.01 highly significant; 0.01 < *p* < 0.05 significant; *p* > 0.05 not significant.

**Table 4:** Effect of extract methods on the yield and the active components’ content in the Gardeniae fructus extract (*n* = 6).

Extract Methods	Extraction Yield, %	Total Iridoids, %	Total Crocin, %	Total Flavonoids, %
HRE	28.97 ± 1.02 ^b^	16.69 ± 0.68 ^b^	2.84 ± 0.11 ^a^	5.98 ± 0.18 ^a,b^
UAE	27.91 ± 1.57 ^b^	16.47 ± 0.90 ^b^	3.00 ± 0.13 ^a^	5.76 ± 0.15 ^b^
MAE	16.73 ± 0.87 ^c^	15.40 ± 0.69 ^b^	2.92 ± 0.25 ^a^	5.83 ± 0.21 ^a,b^
ASE	35.10 ± 0.26 ^a^	27.69 ± 0.85 ^a^	2.35 ± 0.23 ^b^	6.12 ± 0.15 ^a^

Note: ^a,b,c^ mean significant difference between the extract methods (*p* < 0.05).

**Table 5:** Comparison of contents of six characteristic compounds in Gardeniae fructus extracts by different extraction methods (mean ± SD, *n* = 3).

Compounds	HRE (μg/mg)	UAE (μg/mg)	MAE (μg/mg)	ASE (μg/mg)
Geniposidic acid	1.85 ± 0.08 ^b^	2.13 ± 0.07 ^a^	1.77 ± 0.02 ^c^	2.14 ± 0.10 ^a^
Geniposide	160.67 ± 3.48 ^b,c^	165.42 ± 8.17 ^b^	169.41 ± 0.04 ^a^	155.27 ± 1.18 ^c^
Genipin	0.41 ± 0.07 ^b^	0.49 ± 0.02 ^b^	0.45 ± 0.06 ^b^	0.65 ± 0.10 ^a^
Crocin-1	81.49 ± 0.49 ^c^	91.59 ± 3.21 ^b^	99.08 ± 2.53 ^a^	54.10 ± 0.42 ^d^
Crocin-2	1.45 ± 0.09 ^c^	1.65 ± 0.04 ^b^	1.84 ± 0.05 ^a^	0.95 ± 0.07 ^d^
Crocetin	0.11 ± 0.02 ^b^	0.10 ± 0.02 ^b^	0.08 ± 0.01 ^b^	1.30 ± 0.06 ^a^

Note: ^a,b,c,d^ mean significant difference between the extract methods (*p* < 0.05).

**Table 6:** Effects of Gardeniae fructus extracts on T-SOD activity of H_2_O_2_- or Aβ_25–35_-treated PC12 cells (mean ± SD, *n* = 3).

Treatments	HRE (U/mL)	UAE (U/mL)	MAE (U/mL)	ASE (U/mL)
Control	28.28 ± 1.58	28.28 ± 1.58	28.28 ± 1.58	28.28 ± 1.58
H_2_O_2_-treated	9.69 ± 0.97 ^##^	9.69 ± 0.97 ^##^	9.69 ± 0.97 ^##^	9.69 ± 0.97 ^##^
H_2_O_2_-treated + extract 6.25 μg/mg	14.12 ± 0.53 **^,##^	15.20 ± 0.81 **,^##^	19.45 ± 0.88 **^,##^	22.86 ± 0.92 **^,##^
H_2_O_2_-treated + extract 25 μg/mg	18.57 ± 5.11 **^,##^	17.14 ± 0.32 **^,##^	22.62 ± 0.87 **^,##^	23.85 ± 0.86 **^,#^
H_2_O_2_-treated + extract 100 μg/mg	20.86 ± 7.91 **^,##^	18.63 ± 0.74 **^,##^	22.81 ± 0.93 **^,##^	26.52 ± 0.96 **
Control	25.05 ± 1.38	25.05 ± 1.38	25.05 ± 1.38	25.05 ± 1.38
Aβ_25–35_-treated	9.10 ± 1.89 ^##^	9.10 ± 1.89 ^##^	9.10 ± 1.89 ^##^	9.10 ± 1.89 ^##^
Aβ_25–35_-treated + extract 6.25 μg/mg	12.60 ± 1.29 ^##^	13.67 ± 2.90 ^##^	17.94 ± 0.94 **^,##^	20.16 ± 0.91 **^,##^
Aβ_25–35_-treated + extract 25 μg/mg	17.05 ± 1.58 **^,##^	15.62 ± 0.83 **^,##^	20.62 ± 1.39 **^,##^	21.87 ± 0.78 **^,##^
Aβ_25–35_-treated + extract 100 μg/mg	19.35 ± 1.60 **^,##^	17.11 ± 1.02 **^,##^	21.32 ± 0.95 **^,##^	23.66 ± 1.45 **

Note: ^#^ *p* < 0.01, ^##^ *p* < 0.01 compared to control, ** *p* < 0.01 compared to H_2_O_2_- or Aβ_25–35_ peptide-treated groups.

**Table 7:** Effects of Gardeniae fructus extracts on GSH-Px activity of H_2_O_2_- or Aβ_25–35_-treated PC12 cells (mean ± SD, *n* = 3).

Treatments	HRE (U)	UAE (U)	MAE (U)	ASE (U)
Control	45.35 ± 3.96	45.35 ± 3.96	45.35 ± 3.96	45.35 ± 3.96
H_2_O_2_-treated	18.54 ± 1.80 ^##^	18.54 ± 1.80 ^##^	18.54 ± 1.80 ^##^	18.54 ± 1.80 ^##^
H_2_O_2_-treated + extract 6.25 μg/mg	16.87 ± 0.80 ^##^	22.42 ± 2.34 *^,##^	19.85 ± 2.32 ^##^	35.00 ± 2.60 **^,##^
H_2_O_2_-treated + extract 25 μg/mg	27.51 ± 1.84 **^,##^	30.85 ± 1.42 **^,##^	23.17 ± 2.53 **^,##^	39.00 ± 3.09 **
H_2_O_2_-treated + extract 100 μg/mg	34.84 ± 3.20 **^,##^	33.45 ± 2.67 **^,##^	37.57 ± 1.99 **^,##^	41.62 ± 2.36 **
Control	50.10 ± 2.35	50.10 ± 2.35	50.10 ± 2.35	50.10 ± 2.35
Aβ_25–35_-treated	15.36 ± 2.75 ^##^	15.36 ± 2.75 ^##^	15.36 ± 2.75 ^##^	15.36 ± 2.75 ^##^
Aβ_25–35_-treated + extract 6.25 μg/mg	26.22 ± 0.87 **^,##^	23.90 ± 2.62 **^,##^	30.54 ± 4.85 **^,##^	33.32 ± 1.33 **^,##^
Aβ_25–35_-treated + extract 25 μg/mg	30.48 ± 2.22 **^,##^	23.28 ± 0.20 **^,##^	35.09 ± 0.96 **^,##^	37.42 ± 2.42 **^,##^
Aβ_25–35_-treated + extract 100 μg/mg	39.17 ± 3.96 **^,##^	29.18 ± 1.74 **^,##^	36.63 ± 4.08 **^,##^	47.34 ± 2.08 **

Note: ^##^ *p* < 0.01 compared to control, * *p* < 0.05, ** *p* < 0.01 compared to H_2_O_2_- or Aβ_25–35_ peptide-treated groups.

**Table 8:** Effects of Gardeniae fructus extracts on MDA content of H_2_O_2_- or Aβ_25–35_-treated PC12 cells (mean ± SD, *n* = 3).

Treatments	HRE (nmol/mL)	UAE (nmol/mL)	MAE (nmol/mL)	ASE (nmol/mL)
Control	64.36 ± 3.04	64.36 ± 3.04	64.36 ± 3.04	64.36 ± 3.04
H_2_O_2_-treated	109.74 ± 5.59 ^##^	109.74 ± 5.59 ^##^	109.74 ± 5.59 ^##^	109.74 ± 5.59 ^##^
H_2_O_2_-treated + extract 6.25 μg/mg	98.95 ± 3.37 ^##^	102.79 ± 5.78 ^##^	93.38 ± 4.87 *^,##^	84.46 ± 4.86 **^,##^
H_2_O_2_-treated + extract 25 μg/mg	85.28 ± 3.05 **^,##^	81.60 ± 4.05 **^,##^	90.83 ± 1.57 **^,##^	76.42 ± 4.23 **^,##^
H_2_O_2_-treated + extract 100 μg/mg	72.06 ± 4.78 **^,#^	77.70 ± 3.75 **^,##^	80.09 ± 3.19 **^,##^	68.11 ± 4.92 **
Control	61.53 ± 3.35	61.53 ± 3.35	61.53 ± 3.35	61.53 ± 3.35
Aβ_25–35_-treated	107.64 ± 6.29 ^##^	107.64 ± 6.29 ^##^	107.64 ± 6.29 ^##^	107.64 ± 6.29 ^##^
Aβ_25–35_-treated + extract 6.25 μg/mg	95.17 ± 1.46 *^,##^	106.02 ± 2.25 ^##^	96.96 ± 5.82 ^##^	81.15 ± 4.03 **^,##^
Aβ_25–35_-treated + extract 25 μg/mg	80.08 ± 2.40 **^,##^	93.60 ± 1.05 **^,##^	87.52 ± 1.44 **^,##^	73.11 ± 3.92 **^,##^
Aβ_25–35_-treated + extract 100 μg/mg	69.04 ± 5.06 **	83.11 ± 2.04 **^,##^	75.78 ± 0.72 **^,##^	61.80 ± 2.91 **

Note: ^#^ *p* < 0.01, ^##^ *p* < 0.01 compared to control, * *p* < 0.05, ** *p* < 0.01 compared to H_2_O_2_- or Aβ_25–35_ peptide-treated groups.
